# Construction and validation of a nomogram for cancer specific survival of postoperative pancreatic cancer based on the SEER and China database

**DOI:** 10.1186/s12876-024-03180-4

**Published:** 2024-03-13

**Authors:** Wei Peng, Xiaopeng Yu, Renyi Yang, Sha Nie, Xiaolan Jian, Puhua Zeng

**Affiliations:** 1 Affiliated Hospital of Hunan Academy of Traditional Chinese Medicine, Changsha, 410006 People’s Republic of China; 2 School of Integrated Chinese and Western Medicine, Hunan University of Traditional Chinese Medicine, Changsha, 410006 People’s Republic of China; 3 Hunan University of Chinese Medicine, Changsha, Hunan 410208 People’s Republic of China; 4 The Fourth Hospital of Changsha, Changsha, Hunan 410006 People’s Republic of China; 5 Cancer Research Institute of Hunan Academy of Traditional Chinese Medicine, Changsha, Hunan People’s Republic of China

**Keywords:** Postoperative patients with pancreatic cancer, Nomogram, Cancer specific survival, Prediction model, SEER database

## Abstract

**Background:**

The recurrence rate and mortality rate among postoperative pancreatic cancer patients remain elevated. This study aims to develop and validate the cancer-specific survival period for individuals who have undergone pancreatic cancer surgery.

**Methods:**

We extracted eligible data from the Surveillance, Epidemiology, and End Results database and randomly divided all patients into a training cohort and an internal validation cohort. External validation was performed using a separate Chinese cohort. The nomogram was developed using significant risk factors identified through univariate and multivariate Cox proportional hazards regression. The effectiveness of the nomogram was assessed using the area under the time-dependent curve, calibration plots, and decision curve analysis. Kaplan–Meier survival curves were utilized to visualize the risk stratification of nomogram and AJCC stage.

**Results:**

Seven variables were identified through univariate and multivariate analysis to construct the nomogram. The consistency index of the nomogram for predicting overall survival was 0.683 (95% CI: 0.675–0.690), 0.689 (95% CI: 0.677–0.701), and 0.823 (95% CI: 0.786–0.860). The AUC values for the 1- and 2-year time-ROC curves were 0.751 and 0.721 for the training cohort, 0.731 and 0.7554 for the internal validation cohort, and 0.901 and 0.830 for the external validation cohorts, respectively. Calibration plots demonstrated favorable consistency between the predictions of the nomogram and actual observations. Moreover, the decision curve analysis indicated the clinical utility of the nomogram, and the risk stratification of the nomogram effectively identified high-risk patients.

**Conclusion:**

The nomogram guides clinicians in assessing the survival period of postoperative pancreatic cancer patients, identifying high-risk groups, and devising tailored follow-up strategies.

## Background

Pancreatic cancer is a highly fatal disease with poor prognosis. The 5-year survival rate is only 9%, and the incidence rate is still rising steadily [1]. Surgical resection is considered to be the only treatment that can be cured. However, only a few patients with pancreatic cancer are suitable for initial resection. Since pancreatic cancer is usually asymptomatic in the early stage, and most patients are diagnosed as advanced stage [2–4]. Some patients can find the disease during physical examination and undergo early resection, but most patients still relapse and die. Therefore, it is very important to find out the risk factors of postoperative patients with pancreatic cancer and to evaluate the survival prognosis.

In recent years, nomogram has been widely used in tumor prediction, so that clinicians can use it to predict the prognosis of patients [5–7]. A recent investigation has elucidated that a comprehensive analysis encompassing variables such as age, race, histological grade, surgical interventions, and chemotherapy among patients afflicted with bone metastases from pancreatic cancer yields a proficient prediction of survival prognosis. The nomogram's C-index, indicative of model performance, exhibited commendable accuracy [8]. In the study conducted by Wu Mengwei and colleagues, the identification of nine distinctive gene characteristics facilitated the establishment of a prognostic nomogram for the overall survival period in pancreatic cancer. Remarkably, the predictive efficiency surpassed that of the AJCC staging system [9]. Furthermore, the utility of the nomogram has transcended disciplinary boundaries, proving its superior predictive performance over traditional tumor staging methodologies in diverse domains [10–12]. This superiority can be attributed to the nomogram's holistic consideration of a broader spectrum of influential factors.

Nevertheless, investigations concerning postoperative patients with pancreatic cancer remain scarce. Consequently, there exists a critical need for a personalized prediction model tailored specifically to postoperative patients with pancreatic cancer. This imperative underscores our commitment to constructing models aimed at assessing the prognosis and survival rates of individuals post pancreatic cancer surgery.

## Materials and methods

### Patient selection

Patients diagnosed with pancreatic cancer between 2004 and 2015 were initially identified from the SEER database, utilizing SEER * Sta 8.4.0.1 (Surveillance, Epidemiology, and End Results Program at cancer.gov). The external validation cohorts, diagnosed with pancreatic cancer between January 2018 and January 2023, were obtained from the Affiliated Hospital of Hunan Academy of Traditional Chinese Medicine. Inclusion criteria were as follows:Patients with pancreatic cancer who underwent surgery. Availability of clear information on survival status and survival time. Exclusion criteria encompassed:Lack of information on age, sex, marital status, AJCC TNM stage, tumor size, radiotherapy, chemotherapy, and liver metastasis. Patients who died within 1 month or were followed up for less than 1 month after the initial diagnosis. Other causes of death or cases where the cause of death was unknown.

### Cohort definition and variable recode

The entire cohort was randomly divided into training and internal validation cohorts at a ratio of 7:3. The training cohort was employed for risk factor screening and model establishment, while both the internal and external validation cohorts were utilized to validate the results. From the SEER database, 12 variables were screened, encompassing age (at diagnosis), sex, pathological grade, AJCC TNM stage, radiotherapy and chemotherapy status, presence of liver metastasis, tumor size, marital status, and primary site. These variables were crucial in assessing and understanding the factors influencing postoperative survival in patients with pancreatic cancer.

### Statistical analysis

The optimal cut-off values for tumor size and age were determined using X-tile [13]. Univariate and multivariate Cox regression analyses were applied to calculate the corresponding hazard ratios (HR) and 95% confidence intervals (CI) for the training cohort. Independent risk factors identified through these analyses were then incorporated into the nomogram. To assess the nomogram's discriminative ability, the area under the time-dependent curve (AUC value) was calculated. The effectiveness and calibration of the nomogram were evaluated using a calibration curve. The clinical benefit and utility of the nomogram were assessed through decision curve analysis (DCA) [14]. X-tile software was utilized to stratify the risk of the nomogram based on total scores. The Kaplan–Meier method compared the risk stratification of the nomogram with the AJCC stage. Statistical significance was set at *P* < 0.05. All data analyses were conducted using R software in accordance with relevant guidelines and regulations.

## Result

### Survival analysis of postoperative and non-operative patients with pancreatic cancer

**Fig. 1:**
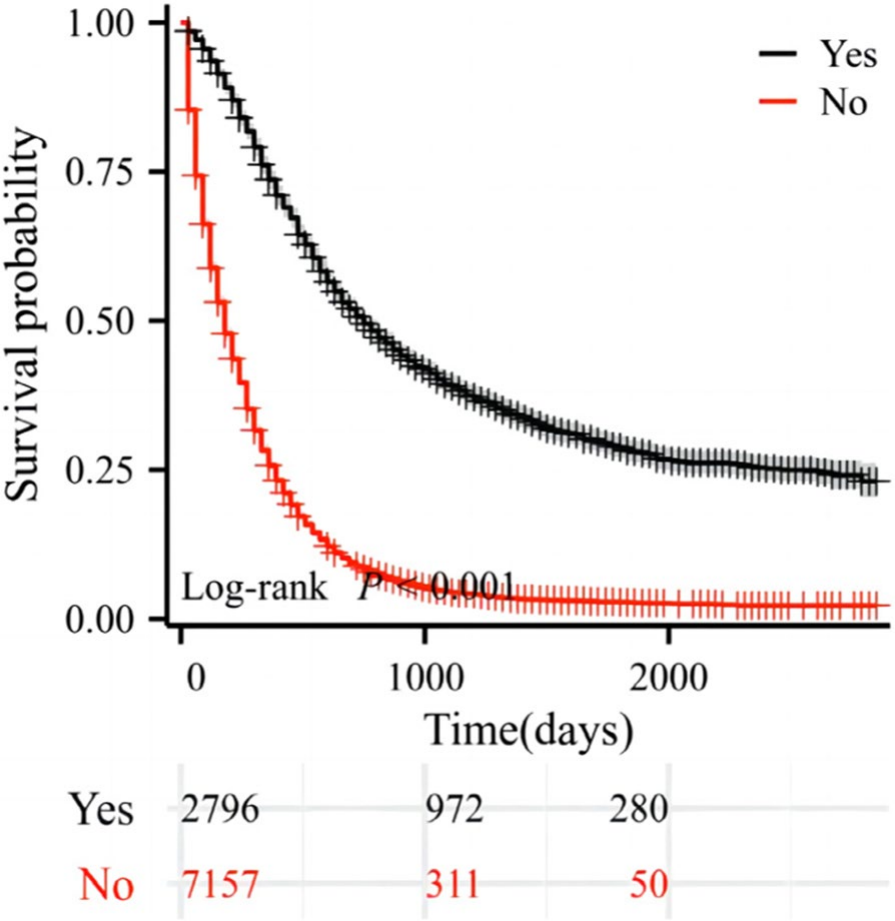
Survival analysis of patients with pancreatic cancer.The black line represents postoperative patients, and the red line represents non-operative patients

In the SEER database, a total of 9953 patients with pancreatic cancer were initially identified. Including 2796 postoperative patients and 7157 non-operative patients. Through Log-rank analysis, the survival possibility of patients who received surgical treatment was significantly better than those who did not receive surgical treatment (*P* < 0.001), as shown in Fig. 1.

### Baseline characteristics of postoperative cancer patients

A total of 2796 postoperative cancer patients were included in this study, comprising 2796 individuals from the SEER database and an additional 71 patients from China. Within the SEER database, patients were randomly partitioned into a training cohorts (*n* = 1957) and an internal validation cohorts (*n* = 839). Simultaneously, the Chinese cases constituted the external validation cohorts (*n* = 71). For detailed information, please refer to Table 1.

**Table 1:** Basic data of postoperative patients with pancreatic cancer

Characteristic	Training	Internal validation	External validation	*P*
n	1957	839	71	
Age, n (%)				< 0.001
< 70	1217 (42.4)	525 (18.3)	60 (2.1)	
≥ 70	740 (25.8)	314 (11)	11 (0.4)	
Sex, n (%)				0.870
Female	943 (32.9)	405 (14.1%)	32 (1.1)	
Male	1013 (35.3)	434 (15.1%)	39 (1.4)	
Grade,, n (%)				0.013
Grade I	292 (10.2)	151 (5.3)	4 (0.1)	
Grade II	839 (29.3)	346 (12.1)	27 (0.9)	
Grade III	583 (20.3)	231 (8.1)	29 (1)	
Grade IV	39 (1.4)	10 (0.3)	0 (0)	
Unknow	204 (7.1)	101 (3.5)	11 (0.4)	
SEER Stage, n (%)				< 0.001
Distant	225 (7.8)	117 (4.1)	55 (1.9)	
Localized	271 (9.5)	132 (4.6)	8 (0.3)	
Regional	1461 (51)	590 (20.6)	8 (0.3)	
T Stage, n (%)				< 0.001
T1	145 (5.1)	68 (2.4)	10 (0.3)	
T2	248 (8.7)	130 (4.5)	27 (0.9%	
T3	1468 (51.2)	590 (20.6)	17 (0.6)	
T4	94 (3.3)	50 (1.7)	15 (0.5)	
TX	2 (0.1)	1 (0)	2 (0.1)	
N Stage, n (%)				< 0.001
N0	746 (26)	338 (11.8)	4 (0.1)	
N1	1210 (42.2)	499 (17.4)	29 (1)	
NX	1 (0)	2 (0.1)	38 (1.3)	
M Stage, n (%)				< 0.001
M0	1831 (63.9)	776 (27.1)	16 (0.6)	
M1	126 (4.4)	63 (2.2)	55 (1.9)	
Radiotherapy, n (%)				<0.001
No	1319 (46)	573 (20)	65 (2.3)	
Yes	638 (22.3)	266 (9.3)	6 (0.2)	
Chemotheropy, n (%)				0.048
No	625 (21.8)	271 (9.5)	13 (0.5)	
Yes	1332 (46.5)	568 (19.8)	58 (2)	
Liver Metastasis, n (%)				<0.001
No	1870 (65.2)	793 (27.7)	39 (1.4)	
Yes	87 (3)	46 (1.6)	32 (1.1)	
Tumor Size, n (%)				<0.001
<19	220 (7.7)	86 (3)	4 (0.1)	
25	1316 (45.9)	581 (20.3)	36 (1.3)	
19–25	421 (14.7)	172 (6)	31 (1.1)	
Marital Status, n (%)				<0.001
Divorce	189 (6.6)	85 (3)	0 (0)	
Married	1310 (45.7)	545 (19)	71 (2.5)	
Single	267 (9.3)	107 (3.7)	0 (0)	
Widow	191 (6.7)	102 (3.6)	0 (0)	
Primary Site, n (%)				<0.001
Other Parts	17 (0.6)	4 (0.1)	5 (0.2)	
Pancreatic Body	205 (7.2)	74 (2.6)	9 (0.3)	
Pancreatic Head	1406 (49)	612 (21.3)	41 (1.4)	
Pancreatic Tail	329 (11.5)	149 (5.2)	16 (0.6)	

### Independent prognostic factors for postoperative patients with pancreatic cancer

**Fig. 2:**
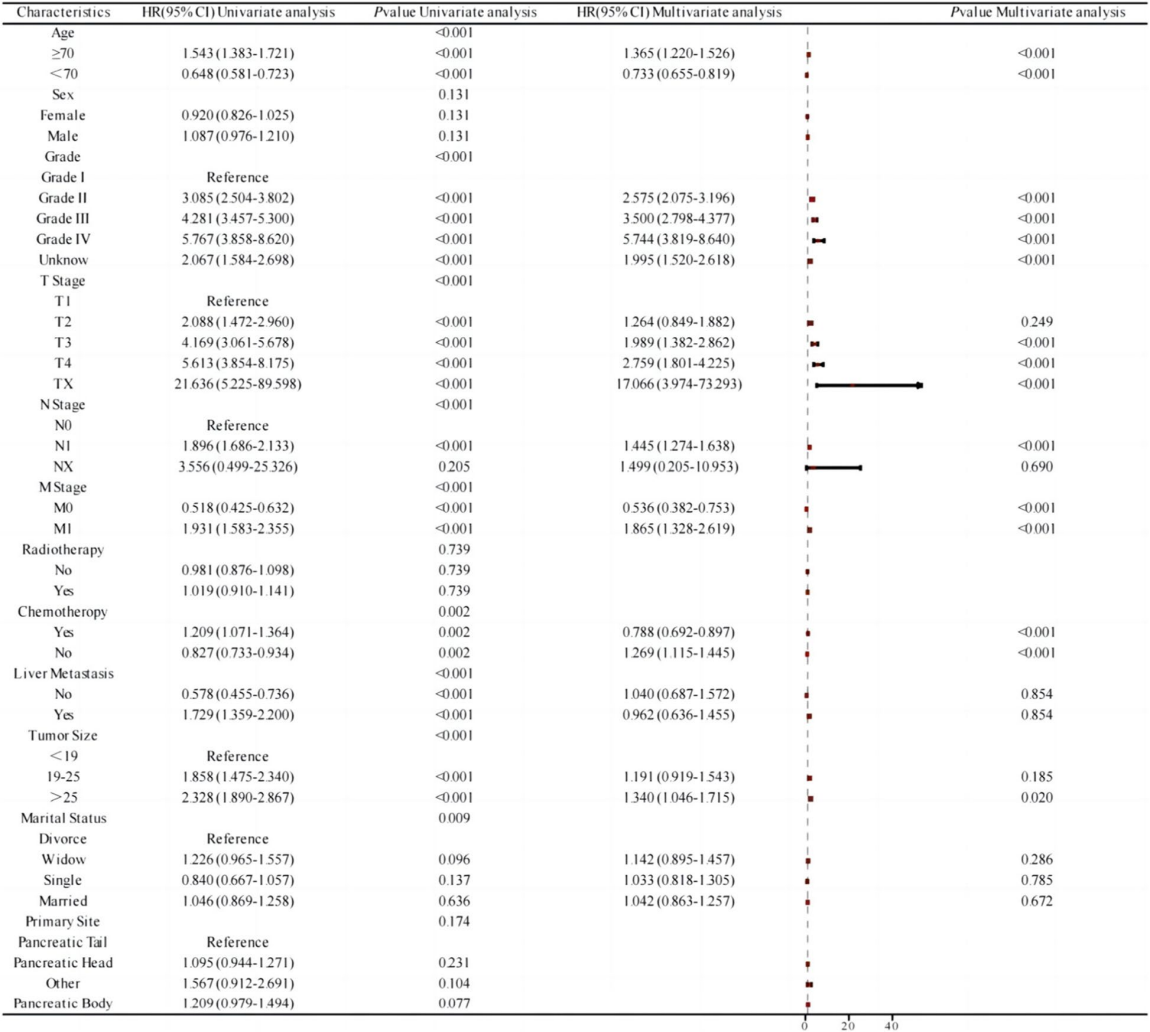
Univariate and multivariate factor analysis forest map

Univariate and multivariate Cox regression analyses were employed to identify independent prognostic factors for postoperative patients with pancreatic cancer. The results revealed that age, pathological grade, chemotherapy, liver metastasis, tumor size (mm), T stage, N stage, M stage, and marital status were prognostic factors for postoperative patients with pancreatic cancer. Specifically, age, pathological grade, chemotherapy, tumor size, T stage, N stage, and M stage were identified as independent prognostic factors influencing cancer-specific survival (CSS), as illustrated in Fig. 2. These findings contribute valuable insights into the multifaceted factors impacting the prognosis of individuals post pancreatic cancer surgery.

### Nomogram construction

**Fig. 3:**
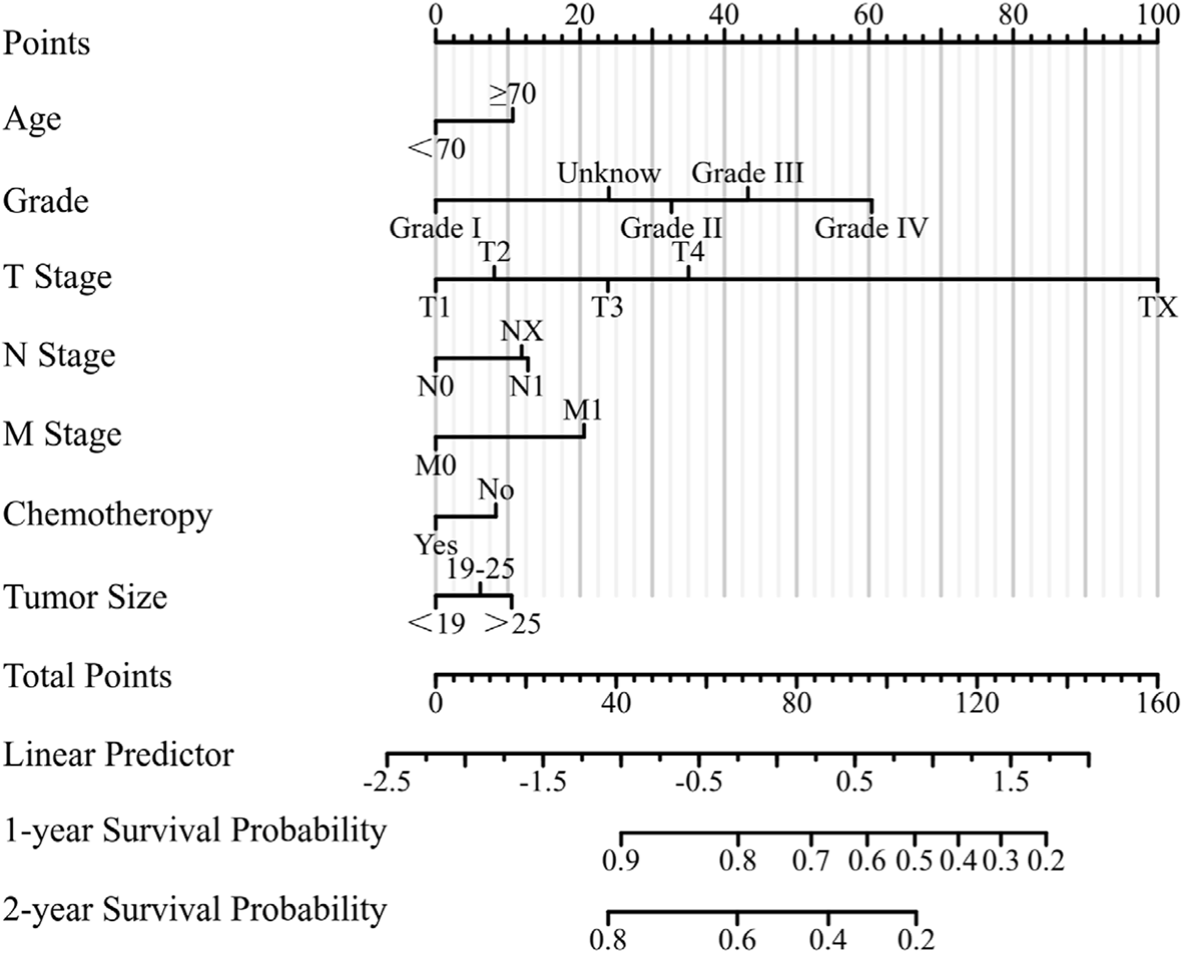
Nomogam of postoperative patient with pancreatic cancer

The prognostic nomogram is based on multivariate Cox regression results. The nomogram (Fig. 3) of 1-, 2-year survival consists of the following independent prognostic factors: age, pathological grade, chemotherapy, tumor size, T stage, N stage and M stage. Each level of these variables was assigned a specific point on the scale. The total score is obtained by adding the scores of each risk factor. In the training, internal validation cohorts and external validation cohorts, the consistency index (C-index) of the nomogram for predicting overall survival (OS) was 0.683(0.675–0.690), 0.689(0.677–0.701) and 0.823(0.786–0.860), respectively. The present study represents a noteworthy advancement in comparison to preceding research endeavors [7, 8].

### Validation of nomogram

#### Time-dependent curves were employed to assess the predictive performance of the nomogram

**Fig. 4:**
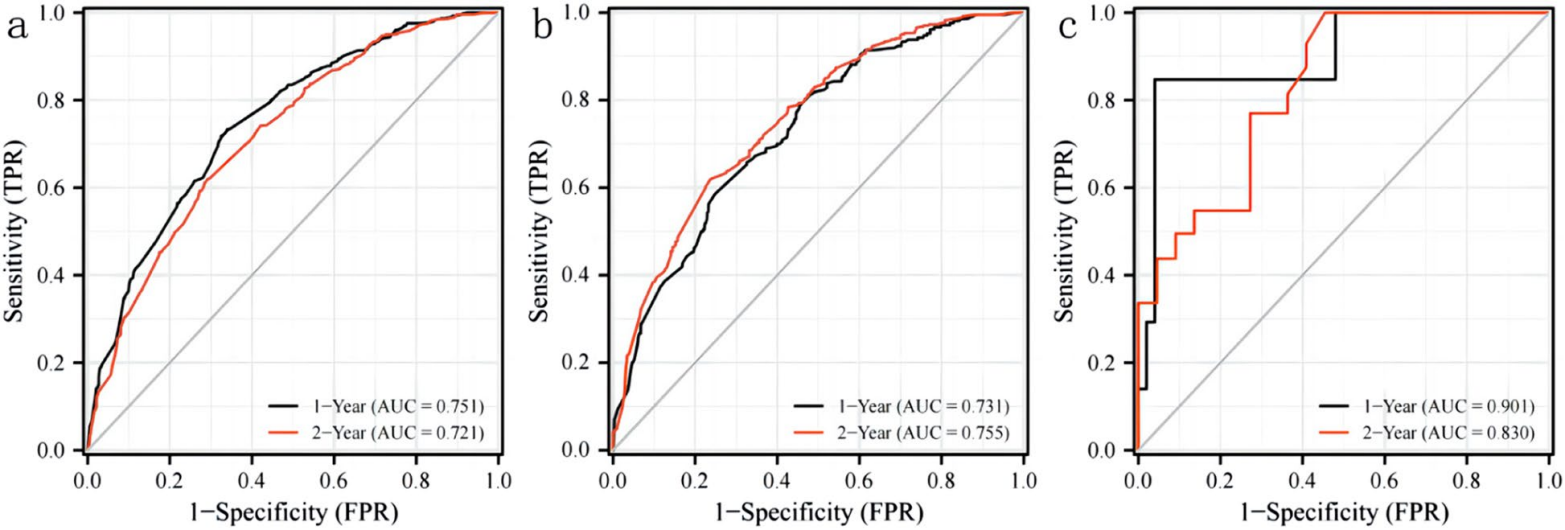
Time-dependent curve. **a** Training cohort; **b** Internal validation cohort; **c**: External validation cohort

**Fig. 5:**
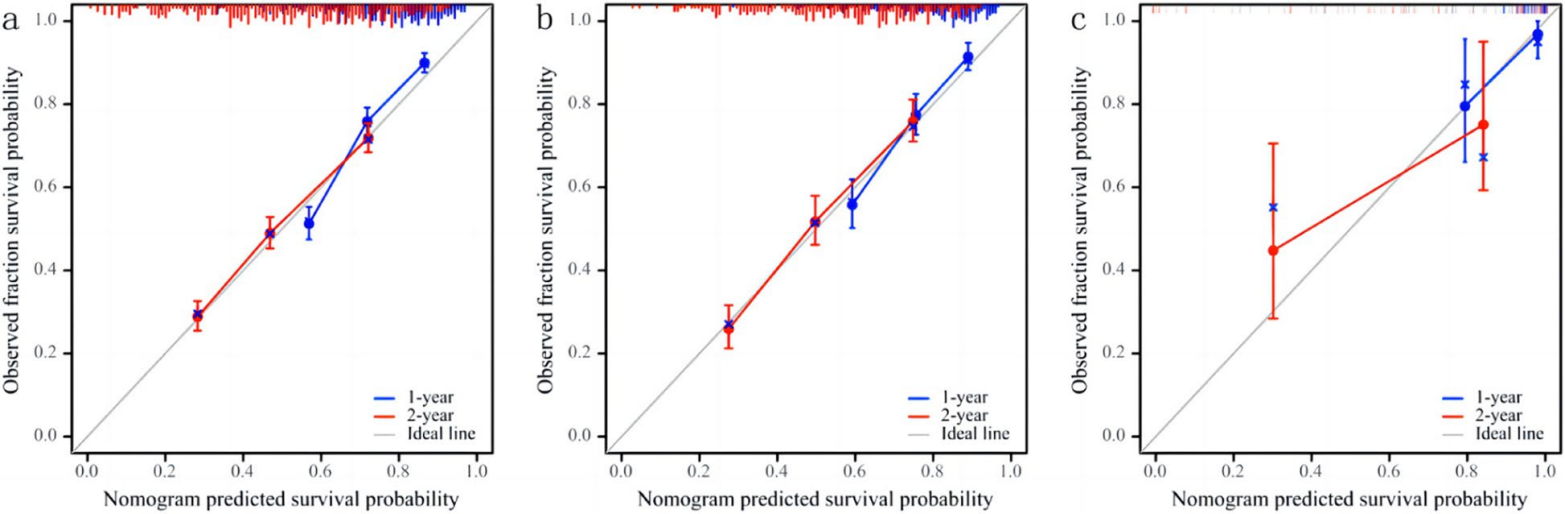
Calibration curve. **a** Training cohort; **b** Internal validation cohort; **c** External validation cohorts cohort. The grey line indicates the ideal reference line where predicted probabilities would match the observed survival rates. Blue line represents 1 year and red line represents 2 years. The closer the blue and red solid lines are to the gray line, the more accurately the model predicts survival

The study indicated AUC values for the training cohort at 1 and 2 years as 0.751 and 0.721, respectively. In the internal validation cohort, these values were 0.731 and 0.755, while in the external validation cohorts, they reached 0.901 and 0.803, respectively. These findings underscored the superior discrimination ability of the nomogram. Refer to Fig. 4 for a visual representation. The calibration curve illustrated a high level of consistency between actual observations and nomogram predictions, as depicted in Fig. 5. This reinforces the reliability and accuracy of the nomogram in predicting postoperative survival outcomes for patients with pancreatic cancer.

#### Validation of decision curve

**Fig. 6:**
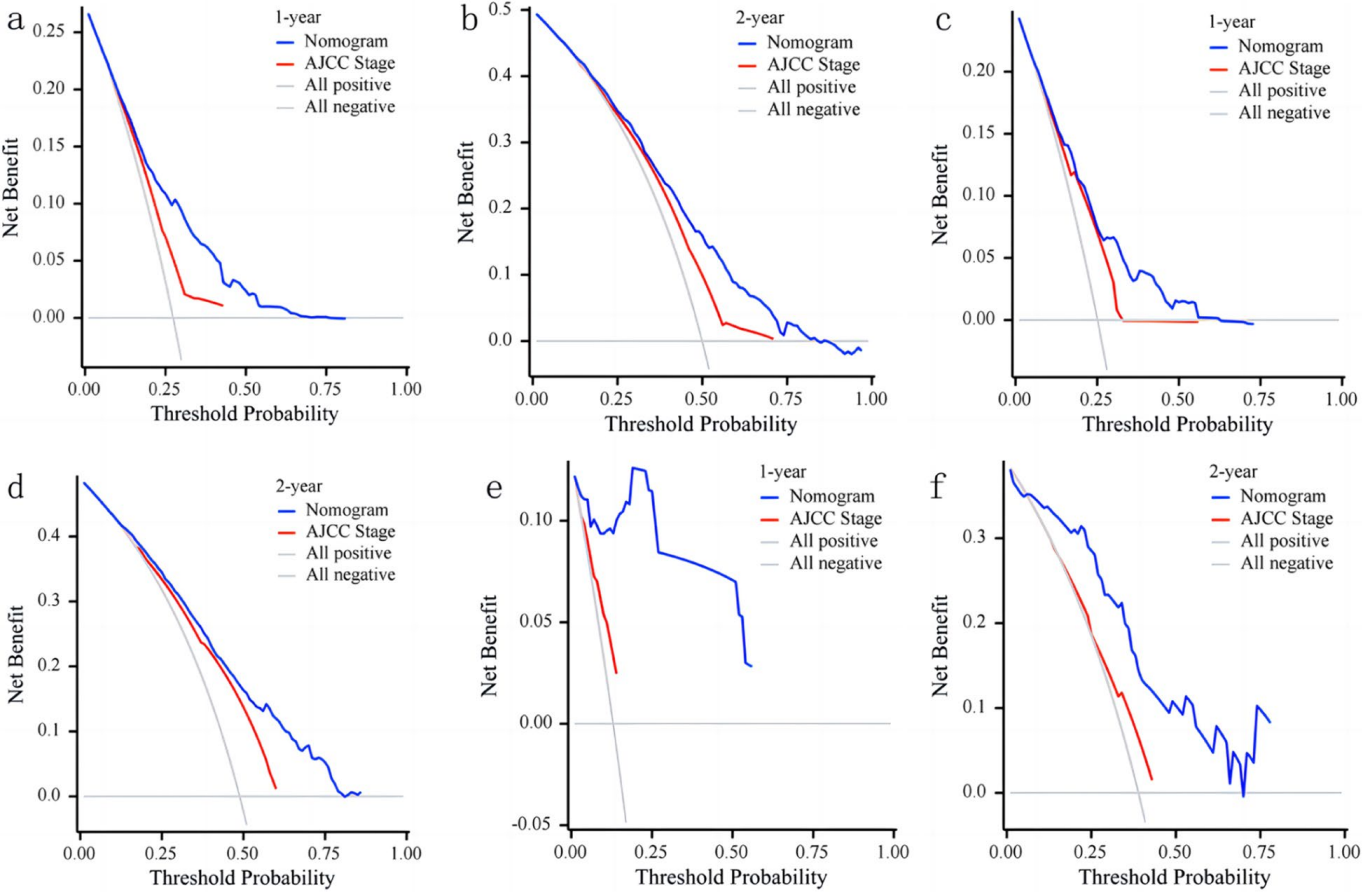
Decision curve analysis of the nomogram and AJCC stage for the CSS prediction of postoperative patients with pancreatic cancer. **a**, **b** 1-and 2-year survival benefit in the training cohort; **c**, **d** 1-and 2-year survival benefit in the internal validation cohort; **e**, **f** 1-and 2-year survival benefit in the external validation cohorts

To assess the clinical benefits, the nomogram was compared with the AJCC stage using Decision Curve Analysis (DCA) curves. The results depicted in Fig. 6 showcased that the nomogram exhibits substantial clinical application potential, yielding a favorable positive net benefit and demonstrating superior clinical practicality compared to the traditional AJCC stage. This emphasizes the enhanced utility of the nomogram in aiding clinical decision-making for postoperative patients with pancreatic cancer.

### Risk stratification of nomogram and AJCC stage

**Fig. 7:**
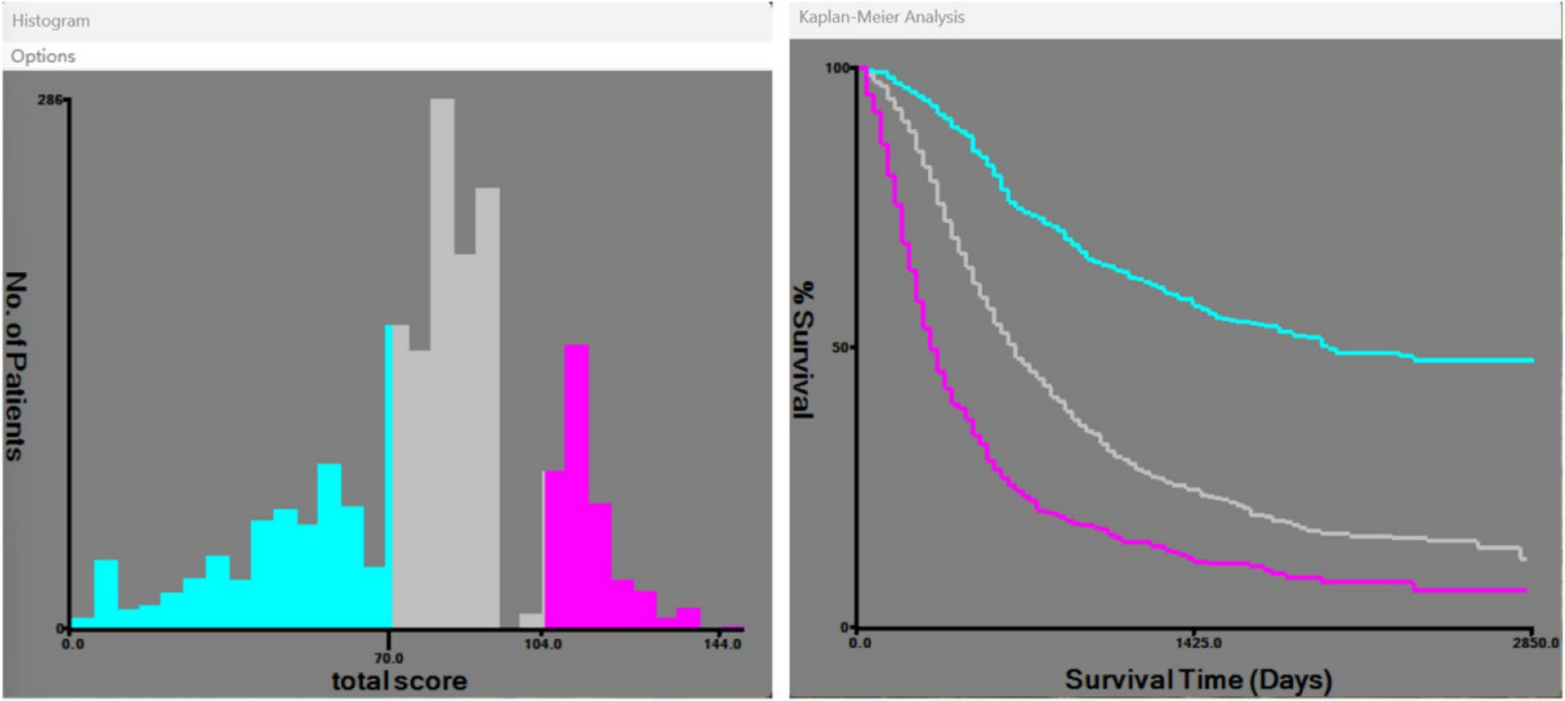
Determination of the cutoff value of training cohorts risk score

The cutoff value of the risk score in the training cohorts is determined by calculating the individual patient scores on the nomogram, ultimately obtaining the total score for each patient. The integration of the patient's survival status, survival time, and total score into X-tile software is performed to derive the cutoff values of risk scores for all patients, as illustrated in Fig. 7. Subsequent internal validation and external cohorts are employed to further assess the efficacy of this cutoff value in distinguishing between different risk levels.

**Fig. 8:**
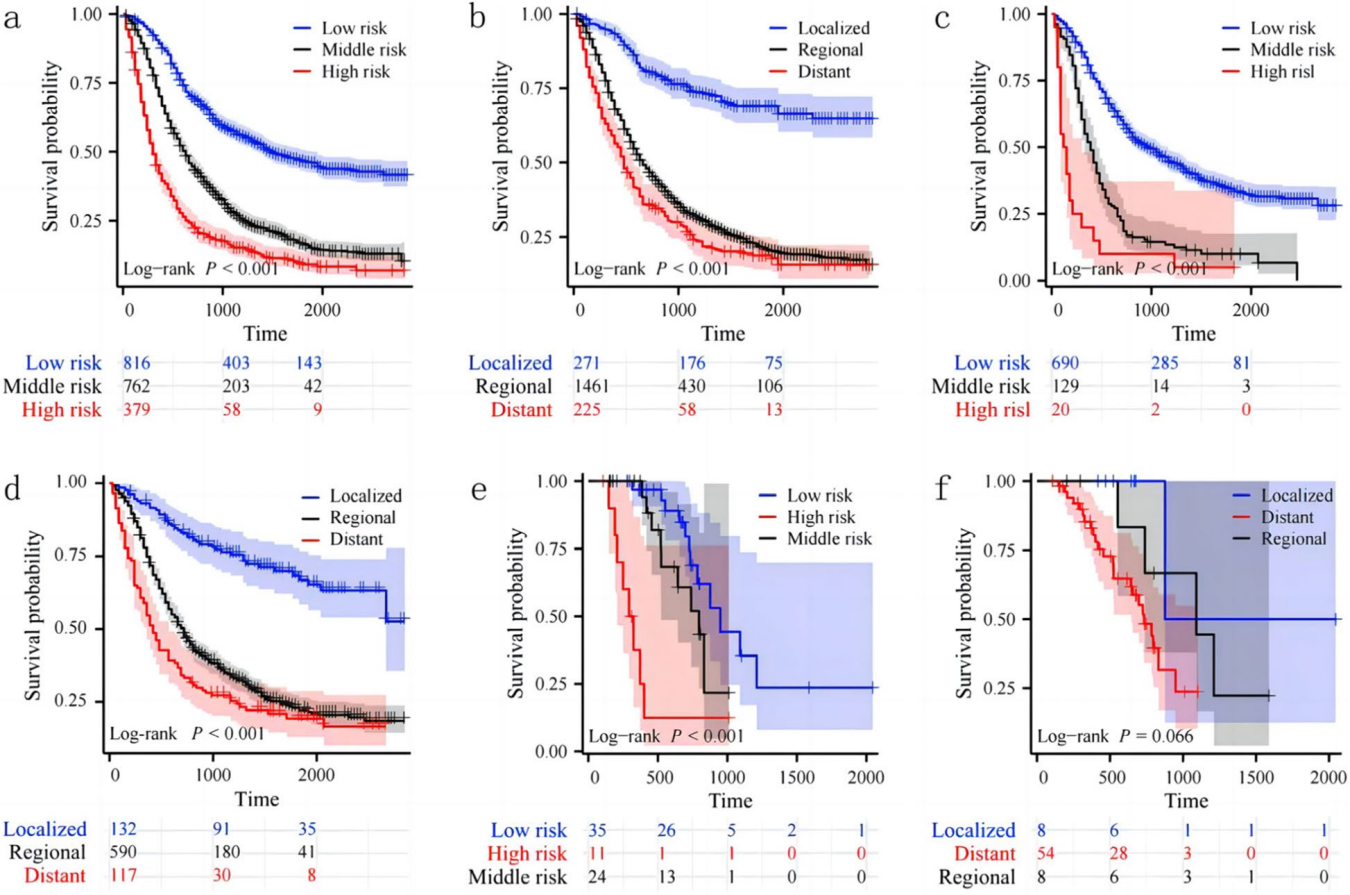
Kaplan–Meier survival curves of postoperative patients with pancreatic cancer at different stages or with different risks stratified by the nomogram. **a** Patients in the training cohort at different risks stratified according to the nomogram; **b** Patients in the training cohort at different stages classified according to the AJCC stage; **c** Patients in the internal validation cohort at different risks stratified according to the nomogram; **d** Patients in the internal validation cohort at different stages classified according to the AJCC stage; **e** Patients in the external validation cohorts at different risks stratified according to the nomogram; **f** Patients in the external validation cohorts at different stages classified according to the AJCC stage

In the final analysis, we stratified the cohort into three risk groups based on the total score derived from the nomogram: low risk (total points < 70), middle risk (total points ≥ 70, < 104), and high risk (total points ≥ 104). The Kaplan–Meier survival curve exhibited notable distinctions among the different risk groups across the entire cohort. Importantly, the nomogram demonstrated superior ability in identifying high-risk individuals compared to the traditional AJCC stage system, as illustrated in Fig. 8. This underscores the enhanced precision and discriminatory power of the nomogram in risk stratification for postoperative patients with pancreatic cancer.

## Discussion

Pancreatic cancer stands out as one of the most invasive and fatally aggressive malignancies. Projections indicate that by the year 2030, it is poised to ascend to the position of the second leading cause of cancer-related fatalities. While radical surgery holds the potential for cancer cure [15], the rates of postoperative recurrence and mortality continue to register high figures [16, 17]. In light of these challenges, the predictive assessment of survival rates among postoperative cancer patients assumes paramount significance.

Several studies have consistently demonstrated that factors such as advanced age, elevated histological grade, and larger tumor size exhibit a negative correlation with long-term survival outcomes [8, 18, 19]. In our investigation, the findings underscore a significant disparity in survival rates between patients who underwent surgical treatment and those who did not. Notably, patients with pancreatic cancer who actively pursued surgical resection exhibited markedly enhanced survival probabilities [20, 21]. This observation aligns with the conclusions drawn by Hester et al., who based on an analysis of the National Cancer Database, established the beneficial impact of surgical resection on the overall survival of pancreatic cancer patients [22]. Nevertheless, reliance on surgery alone is insufficient for achieving prolonged survival. The median survival time for the majority of patients typically hovers around 8 to 10 months, with frequent tumor relapses [23, 24]. Our study incorporated patients receiving chemotherapy, encompassing both preoperative neoadjuvant chemotherapy and postoperative adjuvant chemotherapy. Cox regression analysis identified chemotherapy as an independent risk factor for postoperative pancreatic cancer patients, consistent with prior research [7, 25, 26]. Notably, the median postoperative survival of patients undergoing adjuvant chemotherapy doubled compared to those who did not [27]. Neoadjuvant chemotherapy emerged as an independent predictor and an enhancer of overall survival for postoperative pancreatic cancer patients [28, 29], concurrently improving the R0 removal rate [24, 30]. Consequently, it presents a favorable therapeutic option for both patients and healthcare practitioners. Additionally, age emerged as an independent risk factor for pancreatic cancer patients [8]. Our study revealed a lower survival rate among patients aged 70 years and older. This age-related discrepancy in survival rates may be associated with compromised immunity and physical deterioration commonly observed in elderly patients [18, 19, 31].

The validation of predictive models is crucial for determining generalization and avoiding overfitting [32]. In our investigation, the nomogram exhibited a superior AUC value in comparison to the AJCC staging system, indicative of enhanced discriminative ability. The calibration chart further underscored the robust consistency between the predicted nomogram and the observed 1-year and 2-year cancer-specific survival (CSS), affirming the reliability and repeatability of the established nomogram. Decision Curve Analysis (DCA) analysis reinforced the nomogram's heightened clinical benefits over traditional AJCC staging models. Additionally, nomogram's risk stratification model proficiently categorizes patients into high-risk, medium-risk, and low-risk groups. To our knowledge, this study marks the inaugural utilization of a nomogram for survival prediction, leveraging the SEER database and undergoing external validation, specifically tailored for postoperative cancer patient prognostication. Insights gleaned from our research suggest that characteristics indicative of high-risk status among postoperative cancer patients encompass advanced age, male gender, lower histological grading, larger tumors, and absence of chemotherapy. Crucially, our nomogram surpasses the capabilities and value of the traditional TNM staging system. We contend that meticulously designed nomogram hold the potential to accurately predict the prognosis of each patient, thereby conferring substantial benefits to both clinical practitioners and patients.

This study holds significant clinical importance as nomogram can be employed to assess individualized prognoses in postoperative cancer patients. However, our research is not without limitations. Firstly, being a large-scale retrospective study based on the SEER database, inherent biases associated with retrospective designs cannot be entirely mitigated. Secondly, crucial information related to tumor markers, chemotherapy regimens, and comorbidities is absent from the database, factors known to influence the survival and prognosis of cancer. Lastly, external validation cohorts exclusively comprise the Asian population, with a relatively modest sample size. To validate our research findings, future endeavors should involve prospective clinical trials with expanded sample sizes and diverse ethnic groups. Despite these limitations, our nomogram, rooted in an extensive dataset from the SEER database, offers a robust opportunity to predict cancer-specific survival (CSS) in postoperative patients with pancreatic cancer. This provides valuable support for individualized treatment strategies and more precise clinical decision-making.

## Conclusion

In conclusion, we constructed a nomogram model to assess the cancer specific survival in postoperative patients with pancreatic cancer, which was well validated that it has excellent prediction accuracy. These easy-to-use clinical prediction tools will be useful methods for calculating individualized survival possibility, assisting risk stratification and assisting clinical decision-making for doctors and patients.

## Data Availability

The datasets used and/or analyzed during the current study are available from the corresponding author on reasonable request.
